# P-354. A Case Series of People with HIV Initiating Lenacapavir in Combination with Oral and/or Injectable Antiretrovirals

**DOI:** 10.1093/ofid/ofaf695.572

**Published:** 2026-01-11

**Authors:** Grace Paustian, Marisa B Brizzi, Dimple Patel, Bailey Francis, Carl Fichtenbaum

**Affiliations:** UC Health, Cincinnati, Ohio; UC Health, Cincinnati, Ohio; UC Health, Cincinnati, Ohio; UC Health, Cincinnati, Ohio; University of Cincinnati, Cincinnati, OH

## Abstract

**Background:**

Lenacapavir (LEN) is a first-in-class capsid inhibitor indicated for the treatment of Human Immunodeficiency Virus (HIV) in heavily treatment experienced (HTE) adults in combination with other antiretrovirals (ARVs). Data are limited on the utilization of LEN in the real world setting in combination with injectable and/or oral ARVs.

Table 1. Details of Patients on LEN in Case Series (n= 15)

**Figure:**
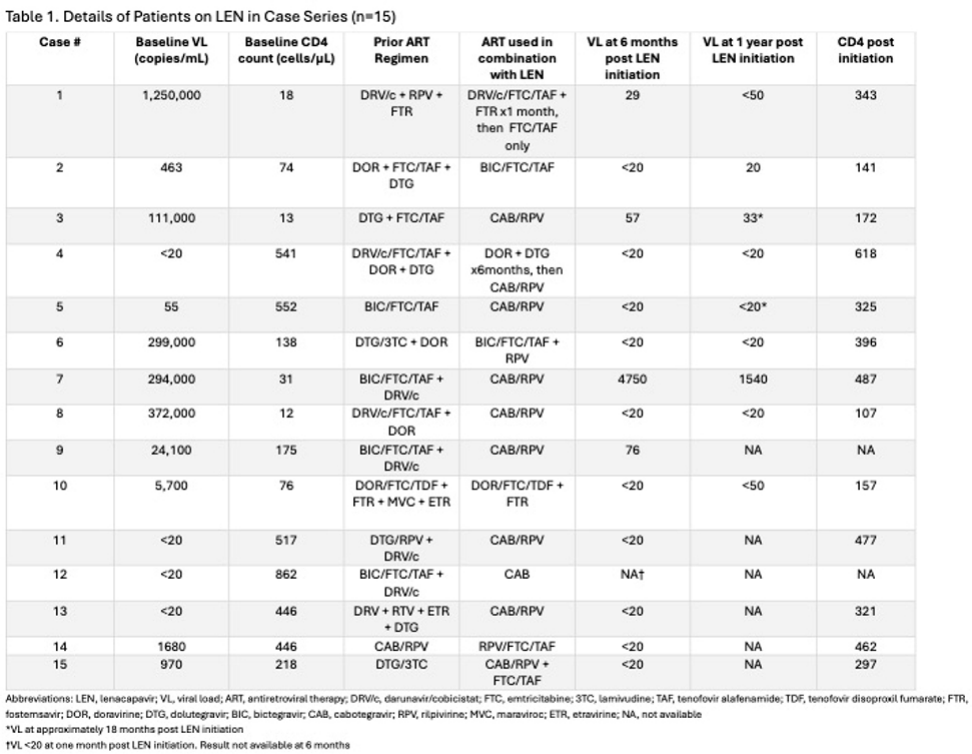

**Methods:**

This retrospective, single-center case series evaluated patients initiated on injectable LEN at UC Health between January 1, 2023, and December 31, 2024. Eligible patients were adults (≥ 18 years) who received at least one injection of LEN with an HIV-1 RNA result at six months post-administration. Primary endpoints included patients who reached or maintained viral suppression (HIV-1 RNA ≤ 50 copies/mL) within six months of switching to LEN-based antiretroviral therapy. Secondary endpoints included the change in CD4 count and reported adverse effects. Descriptive statistics were used for data analysis.

**Results:**

A total of 15 patients were included in the data analysis. The majority of patients included were black (60%), cisgender males (73.3%) with a median age of 52 years. Seven patients (47%) received a combination of injectable LEN and oral medications, while eight (53%) were treated with injectables only. Five patients (33.3%) were virologically suppressed at baseline and ten (66.7%) were not. Within six months of switching to LEN-based ARVs, all five patients who were virologically suppressed at initiation remained suppressed. Of the ten patients not suppressed at initiation, nine achieved viral suppression within six months. The remaining patient’s viral load was 4,700 copies/mL at 7 months post LEN initiation, with baseline viral load of 293,000 copies/mL. At six months, the median CD4 count increased from 175 (12-862) cells/mm³ at baseline to 309 (72-546) cells/mm³ at follow-up with a median duration on LEN therapy of 333 (104-641) days. The most commonly reported adverse effects were injection site pain or soreness.

**Conclusion:**

In this case series of 15 HTE adults with HIV, LEN was associated with high rates of achieving and maintaining viral suppression when used in combination with oral tablets or other injections.

**Disclosures:**

Marisa B. Brizzi, PharmD, BCPS, AAHIVP, Gilead Sciences: Advisor/Consultant|Merck: Advisor/Consultant|ViiV: Advisor/Consultant Bailey Francis, PharmD, BCIDP, AAHIVP, Merck: Honoraria Carl Fichtenbaum, MD, Gilead Sciences: Grant/Research Support|Merck: Grant/Research Support|ViiV Healthcare: Grant/Research Support

